# Complete High-Quality Genome Sequence of Clostridium limosum (Hathewaya limosa) Isolate 14S0207, Recovered from a Cow with Suspected Blackleg in Germany

**DOI:** 10.1128/MRA.01487-19

**Published:** 2020-01-09

**Authors:** Prasad Thomas, Mostafa Y. Abdel-Glil, Anne Busch, Lothar H. Wieler, Inga Eichhorn, Anne Bodenthin-Drauschke, Heinrich Neubauer, Christian Seyboldt

**Affiliations:** a Institute of Bacterial Infections and Zoonoses, Friedrich-Loeffler-Institut, Federal Research Institute for Animal Health, Jena, Germany; b Institute of Microbiology and Epizootics, Department of Veterinary Medicine, Freie Universität, Berlin, Germany; c Robert Koch-Institut, Berlin, Germany; d Landeslabor Schleswig-Holstein, Neumünster, Germany; University of Rochester School of Medicine and Dentistry

## Abstract

Clostridium limosum can be found in soil and the intestinal tract of animals. In 2014, *C. limosum* was isolated from a suspected blackleg outbreak in cattle in Schleswig-Holstein, Germany. We present a complete genome sequence of a *C. limosum* strain represented by a circular chromosome and three plasmids.

## ANNOUNCEMENT

*Clostridium* is a genus of Gram-positive anaerobic bacteria within the phylum *Firmicutes*. The genus includes around 30 species that can cause clinical diseases in humans and animals, including birds. Clostridium limosum is a species that has received little attention in terms of its disease occurrence, prevalence, and virulence factors. The bacterium was found in various environments, including different animal and bird species (1). Recently, it was reported that the pathogen was the principal cause of metritis in farmed minks in Finland (2). In the current study, we isolated *C. limosum* from a suspected blackleg outbreak in cattle from Schleswig-Holstein, Germany. The organism was recovered from liver, spleen, and kidney tissues following anaerobic culture isolation methods. While the morphology on blood agar plates resembled Clostridium chauvoei, PCR results for the detection of *C. chauvoei* and Clostridium septicum (3) remained negative.

An initial categorization of the bacterial species using partial 16S rRNA gene sequencing (4) followed by a BLAST search (https://blast.ncbi.nlm.nih.gov/Blast.cgi; blastn suite; 16S rRNA sequences for *Bacteria* and *Archaea* database) revealed homology to *C. limosum* (Hathewaya limosa Lawson and Rainey 2016 [5]) strain CECT 4329 (NCBI reference sequence number NR_104825).

The *C. limosum* isolate (14S0207) was cultured in 3 ml Selzer broth (6) under anaerobic conditions followed by genomic DNA extraction using a Genomic-tip 100/Q kit (Qiagen, Germany). GATC Biotech (Germany) carried out genome sequencing using a PacBio RS II sequencer (7), including the preceding library preparation to create a 10- to 20-kb insert size library. The total number of reads was 43,854 with an average length of 15,204 bp.

Additional sequencing using paired-end (2 × 300-bp) sequencing technology (MiSeq system) together with the Nextera XT library preparation protocol (Illumina, USA) was performed at the Institute of Bacterial Infections and Zoonoses in Jena, Germany. A total of 1,067,720 reads were received. The raw reads were checked for quality before and after read trimming using FastQC version 0.11.7 (https://www.bioinformatics.babraham.ac.uk/projects/fastqc/). Briefly, reads were trimmed using BBDuk (8) for adaptor removal (with the parameters ktrim=r, k=23, mink=11, hdist=1, tpe, and tbo) and Sickle version 1.33 (9) (with the parameter -q 20) for base quality.

Genome assembly was done using the Hierarchical Genome Assembly Process algorithm version 3 (HGAP3) with default parameters (10) implemented in PacBio SMRT portal version 2.3.0. The seed length obtained during HGAP3 assembly was 10,613 bp (preassembled read length). HGAP3 assembly generated one contig representing the chromosome and three contigs representing the plasmids for the *C. limosum* isolate. The circularization of the received contig to a bacterial chromosome was carried out using a protocol recommended by PacBio for merging and circularization (https://github.com/PacificBiosciences/Bioinformatics-Training/wiki/Circularizing-and-trimming). The overlapping regions of circular sequences were determined using Gepard software version 1.40 with default parameters (11). For the circularization of contigs, Circlator version 1.5.0 was used with default parameters, as described before (12). The circular contigs representing the chromosome and plasmids were initially polished using PacBio long reads with the RS_Resequencing.1 protocol in SMRT portal version 2.3.0 followed by short Illumina reads using Pilon version 1.22 with default parameters (13). As a result, a complete genome is now available that meets high-quality standards. For the PacBio sequence data, we submitted the bax.h5 files and the methylation profiles to NCBI under BioProject number PRJNA432648 and SRA number SRP216188.

The final assembly contained one circular chromosome and three plasmids (Table 1). The annotation was performed with the NCBI Prokaryotic Genome Annotation Pipeline (see Table 1). The chromosome carries a streptolysin-associated gene cluster encoding a streptolysin S (SLS) homolog (locus tag C3495_10690, encoding 52 amino acids), a bacteriocin, and a virulence factor of group A *Streptococcus* known to possess hemolytic/cytolytic activity (14). Studies based on genomic analysis have identified a similar SLS-type gene cluster in *Clostridium* species, including C. botulinum and C. sporogenes, reported as the clostridiolysin S gene cluster (15).

**TABLE 1:** Annotation features of Clostridium limosum 14S0207

Type^*a*^	NCBI RefSeq no.^*b*^	GenBank accession no.	Genome size (Mb)	GC content (%)	No. of proteins	No. of rRNAs	No. of tRNAs	No. of other RNAs	No. of genes	No. of pseudogenes
Chr	NZ_CP026600	CP026600	2.95	28.0	2,527	33	92	4	2,718	62
Plsm	NZ_CP026601	CP026601	0.14	25.5	125				132	7
Plsm	NZ_CP026602	CP026602	0.04	27.5	54				58	4
Plsm	NZ_CP026603	CP026603	0.03	26.4	30				31	1

a Chr, chromosome; Plsm, plasmid.

b RefSeq, reference sequence.

### Data availability.

This whole-genome sequencing project has been deposited at DDBJ/EMBL/GenBank under the accession numbers CP026600 (chromosome), CP026601 (plasmid 1), CP026602 (plasmid 2), and CP026603 (plasmid 3). The raw sequence data are available under SRA accession numbers SRR9822081 (PacBio RS II) and SRR9822082 (Illumina MiSeq). The associated BioProject and BioSample accession numbers are PRJNA432648 and SAMN08456352, respectively. The versions described in this paper are the first versions.
